# Cytogenetic analysis of *Scinax auratus* and *Scinax eurydice* (Anura, Hylidae) with emphasis on cytotaxonomy

**DOI:** 10.3897/CompCytogen.v9i2.4593

**Published:** 2015-05-26

**Authors:** Lídia Nogueira, Fabilene Paim, Débora Diniz, Mirco Solé, Paulo Affonso, Sérgio Siqueira, Iracilda Sampaio

**Affiliations:** 1 Instituto de Estudos Costeiros, Universidade Federal do Pará, Alameda Leandro Ribeiro s/n, Aldeia, Bragança, PA, CEP 68600-000; 2 Departamento de Ciências Biológicas, Universidade Estadual do Sudoeste da Bahia, Rua José Moreira Sobrinho, s/n – Jequiezinho, Jequié - BA CEP: 45200-000; 3 Departamento de Ciências Biológicas, Universidade Estadual de Santa Cruz, Rodovia Ilhéus/Itabuna, km 16, Ilhéus, Bahia, 45662-000, Brazil

**Keywords:** Amphibians, chromosomes, FISH, heterochromatin

## Abstract

*Scinax* Wagler, 1830 is a species-rich genus of amphibians with relatively few detailed chromosomal reports. In this work, cytogenetic analyses of *Scinax auratus* (Wied-Neuwied, 1821) and *Scinax eurydice* (Bokermann, 1968) were carried out based on conventional (Giemsa staining, Ag-NOR and C-banding) and cytomolecular (base-specific fluorochrome staining and fluorescence *in situ* hybridization – FISH of ribosomal probes) techniques. Both species shared the same karyotype, location of active nucleolar organizer regions on pair 11 and GC-rich heterochromatin, as reported for most species in *Scinax ruber* clade. Interpopulation chromosomal variation was observed in *Scinax eurydice*, indicating the occurrence of cryptic species. The mapping of 18S ribosomal genes by FISH is reported for the first time in both species.

## Introduction

Classic and cytomolecular chromosomal studies have been efficient to infer intra and interspecific relationships in anurans, besides supporting the validation of new and cryptic species (Siqueira et al. 2004; Medeiros et al. 2006; Bruschi et al. 2012; Gruber et al. 2012).

The genus *Scinax* Wagler, 1830 encompasses 114 species (Frost 2014), but only 39 of them have been karyotyped (Cardozo et al. 2011) while chromosomal mapping of particular DNA sequences is available solely for *Scinax fuscovarius* (Lutz, 1925) (Kasahara et al. 2003). A review of cytogenetic reports in this genus indicated that all *Scinax* species present a diploid number (2n) of 24 and fundamental number of chromosomal arms (FN) equal to 48. In *Scinax catharinae* clade, the pairs 1 and 2 are submetacentric and nucleolus organizer regions (NORs) in most species are located on pair 6. This pattern differs from *Scinax ruber* clade in which the pairs 1 and 2 pairs are metacentric and the NOR-bearing chromosomes correspond to pair 11 in most species (Cardozo et al. 2011).

*Scinax auratus* (Wied-Neuwied, 1821) inhabits rocky areas in Atlantic forest and forest borders in northeastern Brazil (Alves et al. 2004). This species belongs to *Scinax ruber* clade and, according to biological and anatomical studies would be related to the following species: *Scinax alter* (Lutz, 1973), *Scinax cretatus* (Nunes & Pombal, 2011), *Scinax crospedospilus* (Lutz, 1925), *Scinax cuspidatus* (Lutz, 1925), *Scinax imbegue* Nunes, Kwet & Pombal, 2012, *Scinax juncae* Nunes & Pombal, 2010 and *Scinax tymbamirim* Nunes, Kwet & Pombal, 2012 (Pombal et al. 1995, Alves et al. 2004, Nunes and Pombal 2010, 2011, Nunes et al. 2012, Mercês and Juncá 2012). Cardozo et al. (2011) showed that the karyotype of *Scinax alter* in unique in *Scinax ruber* clade because of a distinctive NOR-bearing pair (3q).

*Scinax euridyce* (Bokermann, 1968) is also widespread in Brazil with records in five states of northeastern and southeastern Brazil (Bahia, Espírito Santo, Minas Gerais, Rio de Janeiro and São Paulo) (Pombal et al. 1995, Hartmann 2002, Canelas and Bertolucci 2007, Araújo et al. 2009, Magrini et al. 2011). Cytogenetic analyses in samples from southeastern Brazil have shown polymorphic NORs since two specimens presented terminal marks on 11q while a single female presented interstitial Ag-NORs (Cardozo et al. 2011).

In the present work, we provide new chromosomal data for both *Scinax auratus* and *Scinax eurydice* in order to respond the following questions: (1) Are the NORs observed in 3q of *Scinax alter* also present in *Scinax auratus*? (2) Is the polymorphism of NORs previously reported in *Scinax eurydice* from southeastern Brazil shared by populations from Bahia? (3) Are there chromosomal differences among geographically distant populations? (4) Can the mapping of 18S rDNA by FISH reveal additional non-active NORs previously undetected by silver nitrate staining?

## Material and methods

Five specimens of *Scinax auratus* and *Scinax euridyce* were collected for cytogenetic analyses in Jequié, state of Bahia, northeastern Brazil (13°51'4"S, 40°4'52"W) (Table 1). Voucher specimens were deposited in the herpetological collection at Universidade Estadual de Santa Cruz – UESC. Mitotic chromosomes were obtained from epithelial cells of intestine as reported by Schmid (1978).

**Table 1.** Analyzed species, number of individuals (N), sex (J = juveniles of undentified sex) and collection site.

Species	Voucher	N	Locality
*Scinax auratus*	MZUESC11051 (♀), MZUESC11052 (♀), MZUESC11053 (♀), MZUESC11054 (♀), MZUESC11055 (♀)	5	Jequié - BA
*Scinax eurydice*	MZUESC11047 (♂), MZUESC11049 (J), MZUESC11005 (J), MZUESC11006 (♂), MZUESC11007 (♂)	5	Jequié - BA

The slides were stained with Giemsa at 10% in phosphate buffer (pH 6.8) for about 10 minutes and air dried. For karyotyping, the chromosomes were classified according to centromere position into: m (metacentric), sm (submetacentric) and st (subtelocentric) following the nomenclature suggested by Green and Session (1991). Active nucleolar organizer regions (Ag-NORs) were detected by silver nitrate staining (Howell and Black 1980) and heterochromatin was visualized by C-banding (Sumner 1972), with slight modifications according to Siqueira et al. (2008). Base-specific fluorochrome with chromomycin A_3_ (CMA_3_), distamycin (DA) and 4,6-diamidino-2-fenilindole (DAPI) was performed to reveal GC- and/or AT-rich sites (Schmid 1980).

Fluorescence *in situ* hybridization using 18S rDNA probes was carried out according to Pinkel et al. (1986), under stringency conditions of 77%. The ribosomal probes were obtained via PCR of genomic DNA of both species (White et al. 1990, Hatanaka and Galetti 2004). In the case of *Scinax eurydice*, the probe was labeled with cyanine 3 (Cy3) by nick translation using Bionick Labeling System kit (Invitrogen) according to manufacturer’s instructions. In *Scinax auratus*, the 18S rDNA probe was labeled using fluorescein-12-dUTP (Roche). The chromosomes were counterstained with DAPI and slides were mounted in Vectashield medium (Vector).

The best metaphase spreads were photographed using an Olympus BX51 epifluorescence microscope equipped with digital image capture system (ImagePro Plus – Media Cybernetics) and processed in the software Adobe Photoshop CS 8.0.1.

## Results

**Figure 1.**
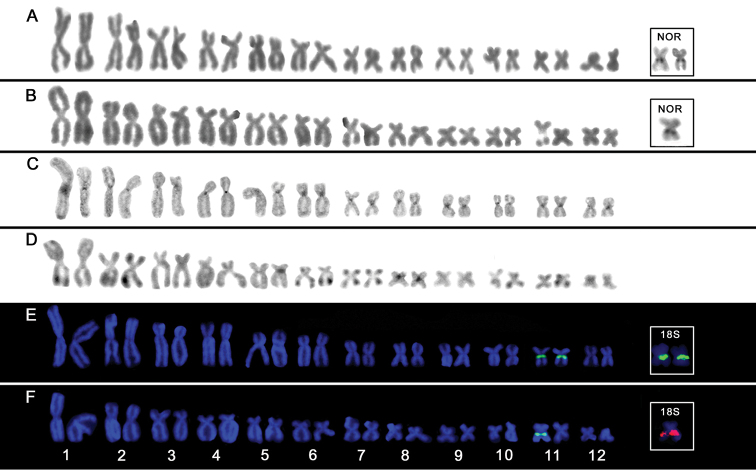
Karyotypes of *Scinax auratus* (**a, c, e**) and *Scinax eurydice* (**b, d, f**) after Giemsa-staining (**a, b**), C-banding (**c, d**) and base-specific fluorochrome staining (**e, f**). The NOR-bearing chromosomes after silver nitrate staining and FISH with 18S rDNA probes of each species are shown in boxes. Bar = 10 µm.

*Scinax auratus* and *Scinax eurydice* presented 2n = 24 and FN = 48 besides sharing the same chromosomal formula: 16 metacentric (pairs 1, 2, 7, 8, 9, 10, 11 and 12) and eight submetacentric (pairs 3, 4, 5 and 6) chromosomes (Table 2; Fig. 1).

**Table 2.** Chromosomal measurements of studied species: relative length (RL), centromeric index (CI) and classification (CT) according to Green and Sessions (1991).

Species	Chromosomal Pairs												
		1	2	3	4	5	6	7	8	9	10	11	12
*Scinax auratus*	RL	16.35 ± 0.09	13.66 ± 0.07	11.40 ± 0.03	10.34 ± 0.55	9.22 ± 0.04	8.73 ± 0.01	6.76 ± 0.26	6.30 ± 0.01	5.48 ± 0.50	5.30 ± 0.01	5.21 ± 0.01	5.11 ± 0.01
	CI	0.49 ± 0.01	0.42 ± 0.01	0.34 ± 0.01	0.36 ± 0.01	0.34 ± 0.01	0.31 ± 0.01	0.45 ± 0.01	0.48 ± 0.01	0.41 ± 0.01	0.38 ± 0.01	0.48 ± 0.01	0.47 ± 0.01
	CP	M	M	SM	SM	SM	SM	M	M	M	M	M	M
*Scinax eurydice*	RL	14.35 ± 0.09	11.52 ± 0.15	10.82 ± 0.43	9.56 ± 0.55	9.01 ± 0.37	7.81 ± 0.21	6.61 ± 0.21	6.45 ± 0.09	5.79 ± 0.60	5.71 ± 0.09	5.56 ± 0.96	4.53 ± 0.15
	CI	0.48 ± 0.01	0.42 ± 0.01	0.27 ± 0.01	0.32 ± 0.01	0.32 ± 0.01	0.3 ± 0.01	0.41 ± 0.01	0.37 ± 0.01	0.43 ± 0.01	0.48 ± 0.01	0.45 ± 0.02	0.47 ± 0.01
	CP	M	M	SM	SM	SM	SM	M	M	M	M	M	M

Silver nitrate staining revealed active nucleolus organizer regions (Ag-NORs) at interstitial region of 11q (Fig. 1a–b, box). However, a single homologous presented silver nitrate marks in *Scinax eurydice*, being coincident with secondary constrictions in all metaphases (Fig. 1b).

Heterochromatin was distributed over centromeric regions of all chromosomes in *Scinax auratus* while telomeric C-bands were observed in most chromosomes of *Scinax eurydice* along with telomeric heterochromatic blocks at centromeric regions of pairs 5 and 8 (Fig. 1c–d). In some metaphases, C-bands were also observed interspersed to NORs at interstitial position of pair 11. After base-specific fluorochrome staining, CMA_3_^+^ signals were detected at NORs in both species, indicating the presence of GC-rich heterochromatin segments (Fig. 1e–f).

FISH with 18S rDNA probes confirmed the single NOR-bearing pair visualized by silver nitrate staining in the analyzed species (Fig. 1e–f, box).

## Discussion

The karyotypes of *Scinax auratus* and *Scinax eurydice* followed the pattern proposed for *Scinax* (2n = 24 and FN = 48). Similarly, the karyotype formulae agree with those reported for species within *Scinax ruber* clade (Faivovich 2002, Kasahara et al. 2003, Cardozo et al. 2011).

Based on morphological traits and vocalization, *Scinax auratus* seems to be closely related to *Scinax alter*, *Scinax cretatus*, *Scinax crospedospilus*, *Scinax cuspidatus*, *Scinax imbegue*, *Scinax juncae* and *Scinax tymbamirim* (Pombal et al. 1995, Alves et al. 2004, Nunes and Pombal 2010, 2011, Nunes et al. 2012, Mercês and Juncá 2012). Karyotypic studies in this group of species are available only for *Scinax alter*, a distinctive species in *Scinax ruber* clade by the presence of terminal Ag-NORs on long arms of pair 3 (Cardozo et al. 2011). Even though *Scinax auratus* and *Scinax alter* shared the same karyotype formulae, the Ag-NORs in the former was identified on pair 11, a plesiomorphic condition reported in most species within *Scinax ruber* clade. Therefore, cytogenetic studies based on mapping of 18S rDNA in closely related species such as *Scinax cretatus*, *Scinax crospedospilus*, *Scinax cuspidatus*, *Scinax imbegue*, *Scinax juncae* and *Scinax tymbamirim* are encouraged to evaluate whether the presence of NORs among the largest pairs is an autopomorphic condition or a synapomorphy of this subclade.

The NORs were associated with CMA_3_^+^ signals in both analyzed species, indicating the presence of GC-rich repetitive DNA interspersed with ribosomal genes, as commonly observed in anurans (Ananias et al. 2007, Campos et al. 2008). In spite of this correlation between base-specific fluorochrome and rDNA, the mapping of 18S rDNA by FISH is necessary to validate the precise location and number of NORs. In the present study, the FISH results confirmed the presence of a single NOR-bearing pair (11q) in analyzed species (Fig. 1e–f). This pattern has been reported in other species submitted to FISH analyses, with exception of *Scinax fuscovarius* whose 18S rDNA signals were mapped onto pair 12 (Kasahara et al. 2003). Nonetheless, Cardozo et al. (2011) stated that the NOR-bearing pair in *Scinax fuscovarius* actually corresponds to the 11^th^ pair, once the smallest chromosomal pairs in *Scinax* are hardly distinguished.

The specimens of *Scinax eurydice* from the state of São Paulo, southeastern Brazil (Cardozo et al. 2011) and those analyzed in the present study had the same karyotype formulae, but different patterns in heterochromatin distribution. While the population from São Paulo presented C-bands at centromeric position in all chromosomes (Cardozo et al. 2011), the population of *Scinax eurydice* from northeastern Brazil showed heterochromatin at terminal regions of most chromosomes and centromeric regions of pairs 5 and 6 only (Fig. 1d). Telomeric C-bands were also reported in other hylids (Kasahara et al. 2003; Busin et al. 2006; Gruber et al. 2012). Similarly, NORs were also differentiated between both populations of *Scinax eurydice* once they were located at interstitial region of a single homologous in pair 11 whereas specimens from São Paulo presented terminal NORs at 11q besides interstitial cistrons in the same chromosome in one female (Cardozo et al. 2011). The physical mapping of 18S rDNA confirmed the location of NORs, even though a single chromosome was marked by FISH.

Other cases of NOR polymorphism have been previously reported in anurans such as *Hyla nana* (Boulenger, 1889) (Medeiros et al. 2006), *Hyla chrysocelis* Cope 1880, *Hyla versicolor* LeConte, 1825 (Willey et al. 1989), *Engystomops petersi* Jiménez de la Espada, 1872 (Lourenço et al. 1998), *Paratelmatobius poecilogaster* Giaretta & Castanho, 1990 (Lourenço et al. 2001), *Scinax alter* and *Scinax hiemalis* (Haddad & Pombal, 1987) (Cardozo et al. 2011). According to some models of evolution of ribosomal genes in eukaryotes as well as experimental evidence in yeasts, the rDNA are tandemly arranged in chromosomes being particularly susceptible to unequal exchanges between sister chromatids (Eickbush and Eickbush 2007). This phenomenon could account for the presence of a larger (and active) cluster of 18S rDNA in one homologue of pair 11in *Scinax eurydice*. Nonetheless, other events such as errors during DNA replication could also lead to this polymorphic NOR state (Amaro-Ghilardi et al. 2008). Apparently, specimens bearing larger amounts of ribosomal DNA have been fixed in the analyzed population either by natural selection (if this NOR phenotype is somewhat adaptive) or by genetic drift.

The presence of heterozygous NORs (Ag^+^/Ag^-^) in *Scinax eurydice* might be related to sex, since this heteromorphic pattern was observed only in males. For instance, females and males of *Gastrotheca riobambae* (Fowler, 1913) were characterized by two and single NOR marks, respectively, mapped on X chromosomes (Schmid et al. 1983). If sex-related NORs are also valid for *Scinax eurydice*, the sex chromosomes in this species would be morphologically homogeneous and further analyses should be carried out to identify putative mechanisms of sex chromosomal determination by other cytogenetic techniques.

Nonetheless, experimental evidence has shown that individuals of salamanders *Plethodon cinereus* (Green, 1818) and *Xenopus laevis* (Daudin, 1802) bearing heterozygous NORs (Ag^+^/Ag^-^), independently on sex, are viable but their fertility is reduced since crosses between heterozygous specimens will produce unviable tadpoles bearing homozygous NORs (Schmid 1982). Therefore, it is possible that fertility of *Scinax eurydice* is also affected by this unusual pattern of NORs what remains to be investigated by inheritance studies in both natural and controlled conditions.

The interpopulation variation of NOR and C-banding pattern among populations of *Scinax eurydice*, associated with slight differences in vocalization between samples from northeastern and southeastern Brazil (Magrini et al. 2011), reinforces the necessity of a taxonomic review of this species.

In conclusion, the detailed cytogenetic characterization of *Scinax auratus* and *Scinax eurydice* showed that *Scinax auratus* shares some chromosomal traits with most of species in *Scinax ruber* clade, but diverges from the putatively closely related *Scinax alter*. The results in *Scinax eurydice* from Bahia revealed differences in chromosomal banding when compared to populations of southeastern Brazil, indicating the presence of cryptic species that should be systematically revised. Therefore, the chromosomal analyses in *Scinax* are potentially useful to both taxonomy and systematics of this group of anurans.
